# The efficacy of alpha‐lipoic acid in the management of burning mouth syndrome: An updated systematic review of randomized controlled clinical trials

**DOI:** 10.1002/hsr2.1186

**Published:** 2023-04-03

**Authors:** Sujan Banik, Antara Ghosh, Hideyuki Sato, Satomi Onoue

**Affiliations:** ^1^ Department of Pharmacy, Faculty of Science Noakhali Science and Technology University Noakhali Bangladesh; ^2^ Laboratory of Biopharmacy, School of Pharmaceutical Sciences University of Shizuoka Shizuoka Japan

**Keywords:** alpha‐lipoic acid, burning mouth syndrome, management, narrative review

## Abstract

**Background and Aims:**

Burning mouth syndrome (BMS) causes burning or uncomfortable feelings in the oral cavity without any obvious injuries. This condition's etiopathogenesis is still unknown, consequently, BMS management is very challenging. Alpha‐lipoic acid (ALA) is a naturally occurring potent bioactive compound that has been found to be useful in the management of BMS in many studies. Therefore, we conducted a comprehensive systematic review to investigate the usefulness of ALA in the management of BMS based on randomized controlled trials (RCTs).

**Methods:**

Different electronic databases, including PubMed, Scopus, Embase, Web of Science, and Google Scholar, were extensively searched to find relevant studies.

**Results:**

This study included nine RCTs that matched the inclusion criteria. In most studies, ALA was given at a dose of 600–800 mg/day, with up to two months of follow‐up. The majority of studies (six out of nine studies) indicated that ALA was more effective in BMS patients than in the placebo‐controlled group.

**Conclusions:**

This comprehensive systematic review provides evidence of the positive outcomes of the treatment of BMS with ALA. However, more research might be needed before ALA can be considered the first‐line therapy for BMS.

## 1 INTRODUCTION

Burning mouth syndrome (BMS) is a chronic condition characterized by discomfort or pain in the oral cavity, especially in the tongue, sometimes in the lips, palate, gums, and buccal mucosa with the absence of any visible mucosal abnormality.^1^ It most frequently occurs in women, especially after menopause at the ages of 50–70 years, and rarely happens to people under 30 years of age.^2^, ^3^ Though the estimated incidence of BMS varies widely, previous studies reported extremely broad ranges of prevalence from 0.7% to 8%, and the prevalence ratio between females and males is about 7:1.^4^, ^5^, ^6^ The etiology of BMS is still not fully understood, but several local (infections and allergic reactions), systemic (nutritional deficiencies and diabetes), and psychological (anxiety and depression) factors have been identified as potentially linked to the condition of BMS.^7^, ^8^, ^9^ Furthermore, BMS is often found to be comorbid with hematological and nutritional deficiencies, and/or psychological problems like anxiety and depression.^10^

The exact pathophysiology of BMS is still not well understood, and there is much debate around this topic in the scientific literature. Scientific evidence suggests that the neuropathic abnormalities in the tiny nerve fibers of the oral mucosa and/or in certain brain regions are associated with a burning sensation in the oral mucosa manifesting as pain, dysgeusia, and xerostomia.^11^ Owing to the limited knowledge of its pathogenesis with a large variety of associated factors, the treatment for BMS is challenging for physicians. According to the literature, different types of drugs, medications, and other interventions have been proposed for the symptomatic treatment of BMS, but there is presently no widely approved regimen available for the management of BMS.^12^ Alpha‐lipoic acid (ALA) is a naturally obtained bioactive compound that has potent antioxidant, anti‐inflammatory, and neuroprotective effects. ALA has the ability to scavenge reactive oxygen species in most parts of the body, both inside and outside of cells, and it is referred to as the universal antioxidant. It is made by the body in very small amounts, and it can also be found in foods like spinach, broccoli, and meats. ALA has been found to be effective in the management of BMS in many studies. Although ALA has reportedly been researched in the treatment of BMS, the outcomes are ambiguous.^13^, ^14^ Therefore, the purpose of this study was to compile a comprehensive summary of the findings of randomized controlled clinical trials that assessed the effectiveness of ALA on BMS.

## 2 METHODS

### 2.1 Literature search strategy

The Preferred Reporting Items of Systematic Reviews and Meta‐Analysis (PRISMA) statement was employed as a standard guideline to conduct this study^15^ (Supporting Information: file 1). To find relevant published studies up to November 2022, we performed an electronic database search in PubMed/Medline, Scopus, Embase, Web of Science, and Google Scholar. The following keywords, such as “lipoic acid, “alpha‐lipoic acid,” “α‐lipoic acid,” “ALA,” “burning mouth syndrome,” “supplementation,” “treatment,” “management,” “prevention,” “clinical trial,” “randomized clinical trial,” “RCT,” and “randomized controlled clinical trial” were used in electronic databases searching. Additionally, the reference lists of all included articles were checked to avoid missing any related studies.

### 2.2 Inclusion and exclusion criteria

The PICOS (population, intervention, comparison, outcomes, and study design) model was utilized to set up the selection criteria (inclusion and exclusion criteria) of this systematic review. The highlighted inclusion criteria of this study were as follows: (i) studies were published in a peer‐reviewed English journal; (ii) the study population was over the age of 18; (iii) the study type was randomized controlled clinical trials; (iv) studies were available in full text (editorial, conference abstracts, and case reports were excluded). Relevant studies published in other languages than English, studies without any control or placebo group, and any observational studies were excluded from this systematic review.

### 2.3 Data extraction

Two authors (SB and AG) independently reviewed included studies and extracted all the data according to the study design and inclusion criteria, and performed a double‐check. The following data were extracted from each study: study characteristics, including first author name, publication year, study design, country, trial duration, dose, route of administration, and sample size; subject characteristics, including participants’ age, sex, and health status; and outcomes. Any inconsistencies, disagreements, and/or differences were resolved through careful discussion with the other authors.

### 2.4 Quality assessment

We assessed the quality of each study based on the Cochrane Collaboration Tool.^16^ This tool consists of the following six items to assess, including studies: (i) the randomization process; (ii) allocation concealment; (iii) participant, personnel, and outcomes; (iv) missing outcome data; (v) selective outcome reporting; and (vi) other possible sources of bias. Overall, the final quality assessments of each score were ranked as good, fair, and unclear.

## 3 RESULTS

### 3.1 Search result

**Figure 1:**
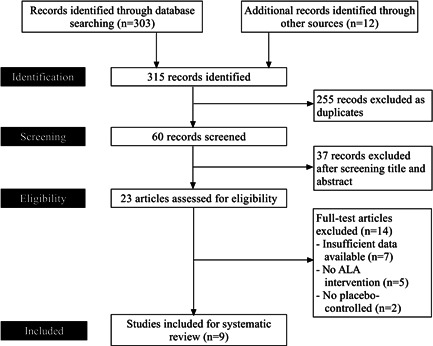
Flowchart showing the literature searching process of the study according to PRISMA guidelines.

Our search approach identified a total of 315 relevant publications. Among them, after removing duplicate records (*n* = 255), and screening by title and abstract (*n* = 37), we selected 23 articles for final evaluation. Nine studies out of 23 publications were finally considered in the present systematic review. Figure 1 depicts the steps used to conduct a literature search, evaluate potential articles, and determine which ones are appropriate for inclusion in the research.

### 3.2 Study characteristics

The demographic study characteristics of the included randomized clinical trials are summarized in Table 1. Four studies were conducted in Italy,^13^, ^17^, ^18^, ^19^ two in Spain,^20^, ^23^ two in Brazil,^21^, ^24^ and one in Argentina^22^ throughout the period from 2000 to 2018. All studies were designed as double‐blinded randomized controlled clinical trials with a sample size of 23 to 120 participants. The mean age of participants was >60 years in most studies, except for two studies.^13^, ^21^ The duration of intervention was between 1 and 2 months, and the dosage of ALA supplementation varied from 600 to 800 mg/day.

**Table 1:** Demographic characteristics of the included studies.

References	Location	Study design	Gender	Mean age (years)	Sample size (n)	Intervention		Main findings
						Treatment group	Control group	
Femiano et al.^17^	Italy	Randomized, double‐blind, placebo‐controlled study	*F* = 32	63	42	600 mg/day ALA for 20 days followed by 200 mg/day for 10 days	Placebo	Significant improvement in ALA treatment group
			*M* = 10					
Femiano and Scully,^18^	Italy	Randomized, double‐blind, placebo‐controlled study	*F* = 42	45	60	200 mg × 3/day ALA for 2 months	Placebo	Significant improvement in ALA treatment group
			*M* = 18					
Femiano et al.,^19^	Italy	Randomized, double‐blind, comparative study	*F* = 28	62	40	Group A: 200 mg × 3/day ALA for 2 months	Group B: 200 mg × 3/day ALA for 2 months with psychotropic therapy	Group A showed greater effectiveness compared to group B.
			*M* = 12					
Carbone et al.^13^	Italy	Randomized, double‐blind, placebo‐controlled study	*F* = 43	67.3	52	400 mg × 2/day ALA for 2 months	Placebo	Lack of efficacy of ALA in BMS
			*M* = 9					
López‐Jornet et al.^20^	Spain	Randomized, double‐blind, placebo‐controlled study	*F* = 54	64.37	60	800 mg/day ALA for 2 months	Placebo	No significant improvement
			*M* = 6					
Cavalcanti and Da Sliveira,^21^	Brazil	Randomized, double‐blind, placebo‐controlled study	*F* = 34	62.9	38	200 mg × 3/day ALA for 1 months	Placebo	Both groups showed improvement, no significant differences between the groups.
			*M* = 4					
López‐D'alessandro and Escovich^22^	Argentina	Randomized, double‐blind, placebo‐controlled study	*F* = 94	57.5	120	600 mg/day ALA for 2 months	Placebo	Significant improvement in ALA treatment group
			*M* = 26					
Palacios‐Sánchez et al.^23^	Spain	Randomized, double‐blind, placebo‐controlled study	*F* = 55	62.13	60	600 mg/day ALA for 2 months	Placebo	Significant improvement in ALA treatment group
			*M* = 5					
Barbosa et al.^24^	Brazil	Randomized, double‐blind, placebo‐controlled study	*F* = 17	60.2	23	200 mg × 3/day ALA for 1 months	Placebo	Significant improvement in ALA treatment group
			*M* = 6					

Abbreviations: ALA, alpha‐lipoic acid; F, female; M, male.

### 3.3 Results of studies

Nine studies reported the efficacy of ALA in the management of BMS (Table 1). In all studies, oral supplementation of ALA was given at a dose of 600–800 mg/day, divided into two or three times daily for two months in most studies. Among nine studies, only two studies did not show any significant improvement in the ALA treatment group^13^, ^20^ and one study reported a level of reduction in burning symptoms for both treatments with no significant differences between the treatment and control group.^21^ The other six studies reported superior improvement in the ALA treatment group in comparison to the placebo‐controlled group.^17^, ^18^, ^19^, ^22^, ^23^, ^24^ Concerning adverse effects, only two studies reported that a few patients had suffered from headaches and abdominal discomfort during the period of ALA treatment.^20^, ^21^

### 3.4 Quality assessment

According to the Cochrane guidelines, most studies were rated with a good quality score, which means reported studies have a low risk of bias (Table 2). All studies followed the randomization process, and outcomes were carefully checked and enlisted in an MS excel sheet.

**Table 2:** Quality assessment using the Cochrane collaboration tool.

References	Randomization process	Allocation concealment	Blinding (personnel, and outcomes)	Statistical test	selective outcome reporting	Overall quality
Femiano et al.^17^	Yes	Yes	Yes	Yes	Yes	Good
Femiano and Scully^18^	Yes	Yes	Yes	No	Yes	Fair
Femiano et al.^19^	Yes	Yes	Yes	Yes	Yes	Good
Carbone et al.^13^	Yes	Yes	Yes	Yes	Yes	Good
López‐Jornet et al.^20^	Yes	Yes	Yes	Yes	Yes	Good
Cavalcanti and Da Sliveira^21^	Yes	Yes	Yes	Yes	Yes	Good
López‐D'alessandro et al.^22^	Yes	Yes	Yes	Yes	Yes	Good
Palacios‐Sánchez et al.^23^	Yes	Yes	Yes	Yes	Yes	Good
Barbosa et al.^24^	Yes	Yes	Yes	Yes	Yes	Good

## 4 DISCUSSION

BMS is a poorly understood chronic condition of the intraoral mucosa owing to its etiology and treatment. It is characterized by discomfort or pain in the absence of a visible lesion to the mouth, but numerous local, systemic, and psychological variables have been identified as potential causes of BMS.^8^, ^9^ Although there is currently no effective protocol for the management of BMS, several studies have found the therapeutic efficacy of ALA in BMS patients, which can be considered a potential treatment option for BMS. ALA is a naturally obtained bio‐active compound that acts as a nutritional cofactor for mitochondrial enzymes and is found to be effective in the treatment of several diseases, including diabetic neuropathy, Alzheimer's disease, and peripheral artery disease, due to its potent free‐radical scavenger activity.^25^ Moreover, it was reported that dihydrolipoic acid (DHLA), a reduced form of ALA, has potential antioxidant activity under in vivo conditions and the ability to regenerate other endogenous antioxidants, such as glutathione (GSH), vitamins E, and C.^26^ In the present study, we summarized the effects of ALA in the management of BMS based on randomized controlled clinical trial reports.

In 2000, Femiano et al. conducted a randomized controlled study for the first time to evaluate the effectiveness of ALA in the treatment of BMS symptoms based on visual analog scale (VAS) scores (worsening, unchanged, slight improvement, decided improvement, and resolution).^17^ They assigned the test subjects into two different groups: the treatment group received 600 mg of ALA/day for 20 days, followed by 200 mg/day for 10 days, and the control group received cellulose starch at a dose of 100 mg/day for 30 days. The outcomes revealed significant improvement in test subjects (76%) after ALA treatment compared with a 14% improvement in the placebo‐controlled group, with no adverse effects in any of the groups. In another study, Femiano and Scully investigated the effectiveness of ALA in BMS patients by giving a dose of 200 mg of ALA three times daily for 2 months compared with the placebo‐controlled group. The results revealed a significant symptomatic improvement in BMS patients with ALA (97%) after 2 months of intervention.^18^ This study suggests that BMS could be a neuropathy linked to the release of toxic free radicals and low levels of GSH from cells when they are stressed. Low levels of GSH may also lead to oxidative stress, inflammation, and nerve damage, which could cause peripheral neuropathy.^27^, ^28^ It has been reported that ALA could help elevate the cellular levels of GSH and be advantageous in the treatment of diabetic polyneuropathy pain and paresthesia by preventing nerve fiber degeneration. Thus, it is predicted that ALA would have a strong influence on the management of BMS. The other four studies also tested ALA versus placebo and reported significant positive outcomes in the management of BMS compared to the placebo‐treated group (Table 1).

Multiple pharmacological treatment options, including antidepressants, anticonvulsants, and antipsychotics, have been reported in the literature for the management of BMS. Among them, topical or systemic antidepressant therapy is a popular treatment option for the symptomatic improvement of BMS because it is considered a psychosomatic condition.^29^ Therefore, Femiano et al. studied the effects of ALA in BMS patients who had taken antidepressants for about 6–12 months (group A) compared with those who had not undergone psychiatric treatment (group B).^19^ The outcomes of the study revealed greater effectiveness of ALA in BMS patients who had not received any antidepressants. In another study, López‐D'alessandro et al. evaluated the action of ALA in comparison to gabapentin (GABA) on the symptoms of BMS.^22^ GABA is an anticonvulsant, previously used by White et al. to improve the symptoms in BMS patients, and they concluded that GABA has little effect on the treatment of BMS.^30^ López‐D'alessandro et al. also reported that the efficacy of ALA was better than that of GABA for reducing burning in patients with BMS, but the most favorable results were reported with the administration of both drugs.

In contrast, Carbone et al. performed a double‐blind, placebo‐controlled trial with 52 patients who received 400 mg of ALA twice daily to examine the effectiveness of ALA in the treatment of BMS and found that ALA was ineffective.^13^ Similarly, López‐Jornet et al. utilized the same concentration of ALA as a placebo and found no significant changes between the groups when comparing ALA to a placebo.^20^ They didn't have a careful explanation of why their results were different from other research, but they believed that the dose should be unified for the management of BMS, while they administered 800 mg/day of ALA orally for 2 months, others used 600 mg/day for 2 months. Concerning side effects, there were no major adverse effects reported in any study.

Despite the importance of this systematic review, there are several limitations to this study. First, the included studies and sample sizes in each study were small and only tested to alleviate BMS symptoms. Secondly, the included studies did not evaluate any other outcomes such as quality of life, psychological status, any marker of BMS, or social functioning. Lastly, we could not do any meta‐analysis to combine the results of the studies because of the lack of data amenable to meta‐analysis and a wide variety of trial methods for the assessment of ALA in the management of BMS.

## 5 CONCLUSION

This systematic review comprehensively investigated the effects of ALA in the management of BMS based on VAS findings, and the majority of studies revealed positive outcomes in the management of BMS with ALA. There have been a few studies that have indicated the outcomes of treatment and placebo groups are nearly identical. However, further research with larger samples is needed before ALA may be regarded as a first‐line treatment option for BMS patients.

## AUTHOR CONTRIBUTIONS

**Sujan Banik**: Conceptualization; supervision; writing—original draft; writing—review and editing. **Antara Ghosh**: Formal analysis; methodology; writing—review and editing. **Hideyuki Sato**: Formal analysis; investigation; methodology; writing—review and editing. **Satomi Onoue**: Conceptualization; supervision; visualization; writing—review and editing.

## CONFLICT OF INTEREST STATEMENT

Sujan Banik is an Editorial Board member of Health Science Reports and a corresponding author of this article. He was excluded from all editorial decision‐making related to the acceptance of this article for publication in this journal. The other authors have declared that they have no competing interests.

## TRANSPARENCY STATEMENT

The lead author Sujan Banik affirms that this manuscript is an honest, accurate, and transparent account of the study being reported; that no important aspects of the study have been omitted; and that any discrepancies from the study as planned (and, if relevant, registered) have been explained.

## Supporting information


**Supplementary file 1**: PRISMA‐2020 Guideline's checklist.

## Data Availability

All data relevant to the study are included in the article.
